# Light rare earth element depletion during *Deepwater Horizon* blowout methanotrophy

**DOI:** 10.1038/s41598-017-11060-z

**Published:** 2017-09-04

**Authors:** A. M. Shiller, E. W. Chan, D. J. Joung, M. C. Redmond, J. D. Kessler

**Affiliations:** 1 Center for Trace Analysis, University of Southern Mississippi, Stennis Space Center, Mississippi 39529 United States; 2 Earth and Environmental Sciences, University of Rochester, Rochester, NY 14627 USA; 3 Department of Biological Sciences, University of North Carolina at Charlotte, Charlotte, NC 28223 USA

## Abstract

Rare earth elements have generally not been thought to have a biological role. However, recent work has demonstrated that the light REEs (LREEs: La, Ce, Pr, and Nd) are essential for at least some methanotrophs, being co-factors in the XoxF type of methanol dehydrogenase (MDH). We show here that dissolved LREEs were significantly removed in a submerged plume of methane-rich water during the *Deepwater Horizon* (*DWH*) well blowout. Furthermore, incubation experiments conducted with naturally methane-enriched waters from hydrocarbon seeps in the vicinity of the *DWH* wellhead also showed LREE removal concurrent with methane consumption. Metagenomic sequencing of incubation samples revealed that LREE-containing MDHs were present. Our field and laboratory observations provide further insight into the biochemical pathways of methanotrophy during the *DWH* blowout. Additionally, our results are the first observations of direct biological alteration of REE distributions in oceanic systems. In view of the ubiquity of LREE-containing MDHs in oceanic systems, our results suggest that biological uptake of LREEs is an overlooked aspect of the oceanic geochemistry of this group of elements previously thought to be biologically inactive and an unresolved factor in the flux of methane, a potent greenhouse gas, from the ocean.

## Introduction

The April 20, 2010 *Deepwater Horizon* (*DWH*) blowout of the MC252 Macondo well in the northern Gulf of Mexico (28° 44′ 17.3″N, 88° 21′ 57.4″W) resulted in the worst oil spill in US history. While the exact amount of hydrocarbon release has been a matter of some discussion^1–4^, approximately 780,000 m^3^ of crude oil and 10^10^ moles of natural gas were released to the environment during the 87 day event. Nearly all of the gas and roughly half of the oil accumulated in submerged plumes between 900 and 1200 m water depth^4–6^. Methane concentrations, for example, ranged up to near millimolar levels^7^, some five orders of magnitude higher than background concentrations^8, 9^. Modeling of dissolved oxygen anomalies at these depths^10, 11^ led to the realization that a bloom of bacteria^12, 13^ rapidly consumed most of the hydrocarbons in these plumes. This has also raised the question of whether the availability of nutrients limited the rate of hydrocarbon consumption at depth^13–15^. Unfortunately, only a few observations of macro- and micro-nutrient concentrations during the *DWH* blowout have been reported. For instance, in surface waters contaminated with *DWH* crude oil, there was evidence of hydrocarbon-supported respiration but with only limited microbial growth due to low nutrient concentrations^16^. However, in the deep microbial bloom, while macronutrients (nitrate and phosphate) were measurably removed, they were likely not limiting^14^. The key trace elements, Fe and Cu, were not depleted in the deep waters^15^, though it is possible that ambient concentrations of these metals were so low as to functionally limit growth^13^. Thus, the issue of deep water nutrient limitation during the blowout remains uncertain.

With regard to bacterial consumption of the *DWH* methane, methanotrophs can utilize a soluble or particulate methane monooxygenase enzyme (sMMO or pMMO) to convert CH_4_ to methanol during the first step of aerobic methane oxidation. After this initial oxidation step, methanol is oxidized to formaldehyde by a methanol dehydrogenase (MDH) enzyme^17–19^. This MDH is often Ca(II)-dependent (MxaF type). However, recent work in volcanic mudpots^20^ has indicated that light rare earth elements (LREEs, including La, Ce, Pr, and Nd) are essential for at least some methanotrophs as cofactors in an alternative form of methanol dehydrogenase (XoxF type; refs 20 and 21) that is also found in oceanic waters^22–24^. Trivalent lanthanides have ionic radii, coordination, and ligand preferences similar to Ca^2+^ ^20^. Thus, the lanthanides can function similarly to Ca ions in some enzymes where they can also be more efficient hydrolytic catalysts because they are stronger Lewis acids than Ca^2+^ ^25^. The reason that lanthanide variants of metallo-enzymes are not more prevalent is likely due to the low dissolved concentrations of lanthanides in most natural waters, especially relative to Ca^25^. Nonetheless, given that oceanic dissolved REEs are in the picomolar concentration range^26, 27^, the observation of *xox*F genes or proteins in the marine environment^22–24^ raises the possibility that availability of LREEs might have limited or altered the mechanism^28^ of *DWH* methanotrophy as well as altered the LREE distributions. Accordingly, we reanalyzed for REEs several sets of dissolved trace element samples previously collected from the vicinity of the wellhead during and after the *DWH* event^15^. We also conducted incubation experiments with naturally methane-enriched waters from hydrocarbon seeps in the general vicinity of the *DWH* wellhead in order to further examine the possible role of LREEs in oceanic methanotrophy. Our combined trace element and metagenomic work lends insight into both the biological role of LREEs as well as the abundance of various bacteria during the *DWH* blowout. We also consider the related questions of whether availability of LREEs could play a role in the fate of massive methane release from hydrate destabilization^29^ and whether water column methanotrophy might alter oceanic LREE distributions.

## Results and Discussion

**Figure 1:**
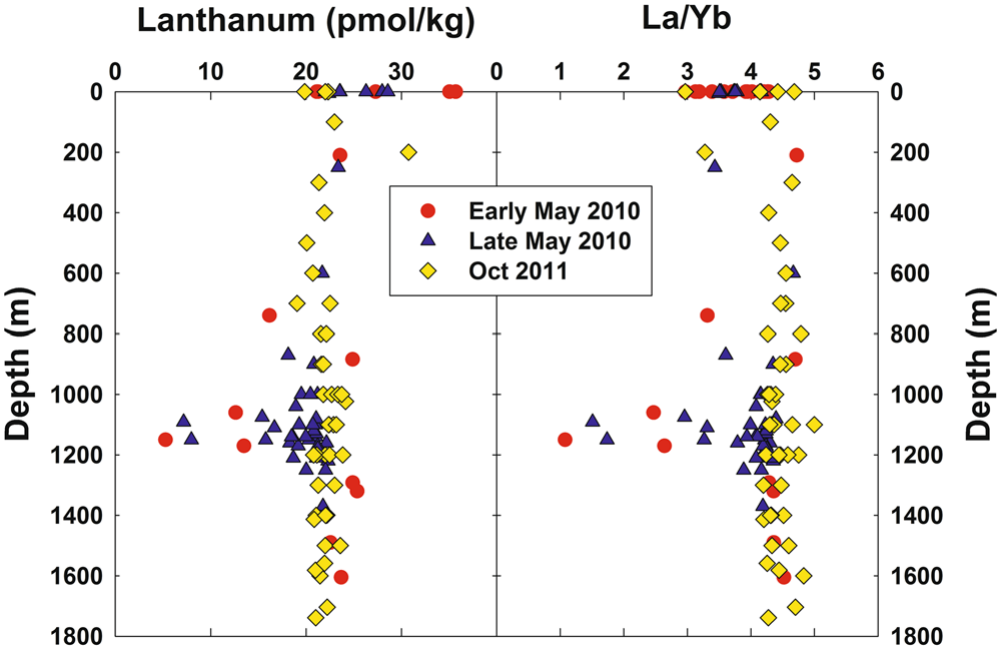
Vertical distributions of dissolved lanthanum and the La/Yb molar ratio in samples collected in the vicinity of the *Deepwater Horizon* site in early and late May 2010 and October 2011. Methane-enriched waters were observed between 1000–1200 m during the 2010 blowout^7, 10^.

**Figure 2:**
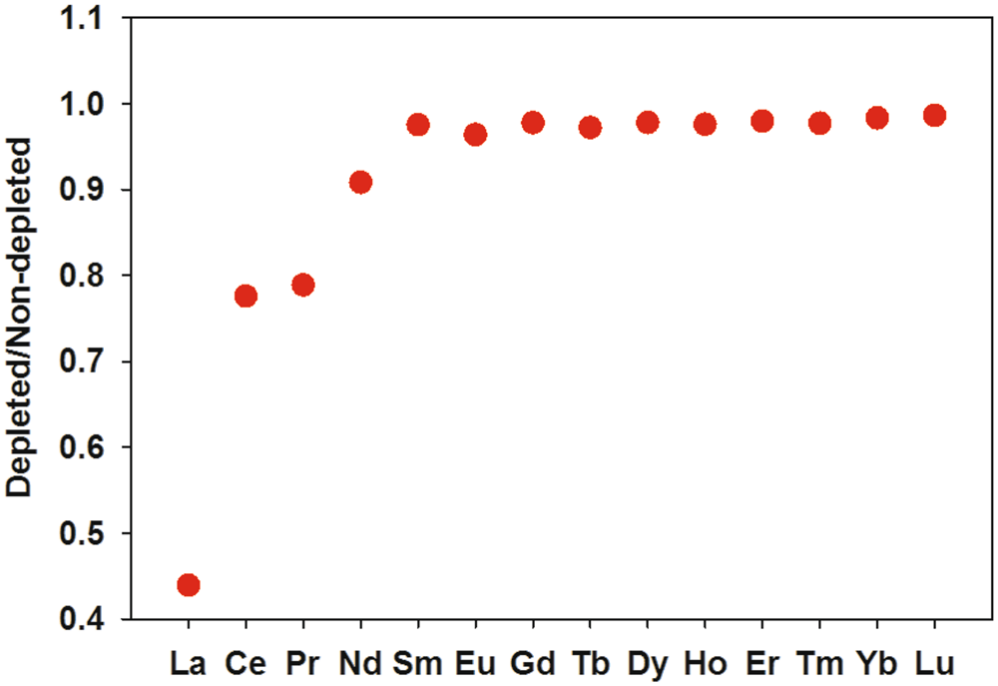
Average ratio of dissolved REE concentrations in the five most LREE-depleted samples to the mean deep water concentrations of non-depleted samples, May 2010.

Figure 1 shows composite profiles of dissolved La and the La/Yb ratio in the vicinity of the *DWH* wellhead. During May 2010 (i.e., during the blowout), some samples in the depth range of the submerged gas/oil plumes were clearly depleted in dissolved La and this depletion affected light but not heavy REEs, as exemplified by the anomalies in the La/Yb ratio (Fig. 1). Outside of the depth range of submerged plume waters, REE concentrations were similar to typical North Atlantic Ocean concentrations^26, 27^, except for some low-salinity surface waters which were affected by the higher dissolved REE concentrations of Mississippi River water^30^. For the five most La-depleted samples, dissolved La concentrations were typically 40% depleted relative to water column samples outside of the submerged plume. Three other LREEs (Ce, Pr, and Nd) also showed measurable (>10%) concentration depletions in the La-depleted samples (Fig. 2). In contrast, during October 2011 (over a year after the blowout was stopped), no LREE concentration depletions were observed (Fig. 1). Thus, the LREE depletions we observed seem clearly linked with the submerged hydrocarbon plumes during the *DWH* blowout.

**Figure 3:**
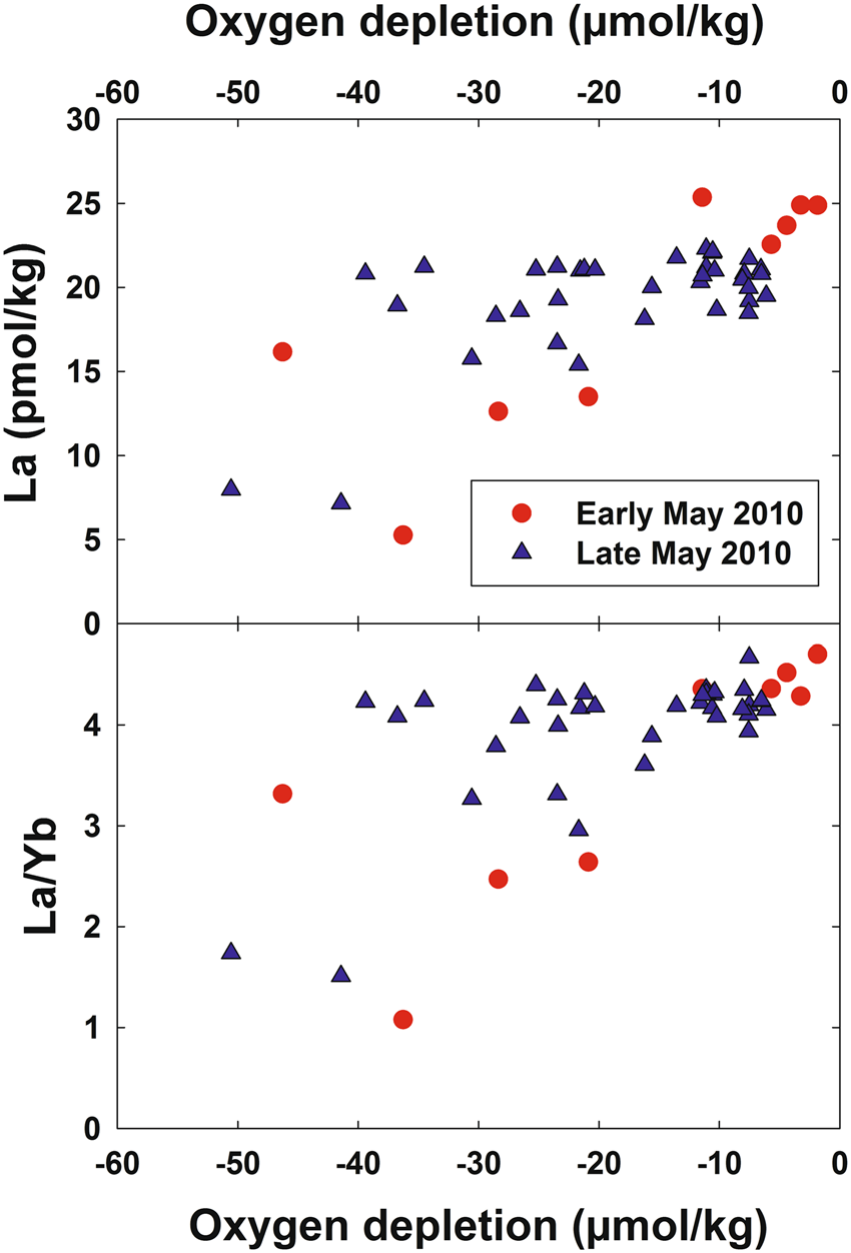
Dissolved La and the La/Yb molar ratio versus oxygen depletion in deep water samples collected near the *Deepwater Horizon* site.

Observation of depletions in La, Ce, Pr, and Nd near the *DWH* wellhead is compatible with Pol *et al*.’s^20^ work showing these four LREEs supported growth of an acidophilic methanotroph in volcanic mudpots, but that higher atomic weight REEs were less conducive to growth. Furthermore, Keltjens *et al*.^21^ showed that the LREE-utilizing XoxF type of MDH is common in methanotrophs. For the late May 2010 cruise, published methane data (sampled on different hydrocasts from the trace element samples) show methane to have been generally highly enriched at depths where we observed LREE depletion^13^. We also observed that the most La-depleted samples had measurable depletions in dissolved oxygen (Fig. 3). Du & Kessler^11^ estimated that these anomalies in dissolved oxygen were largely the result of methanotrophy. Thus, association of the LREE depletions with methanotrophy seems likely.

**Figure 4:**
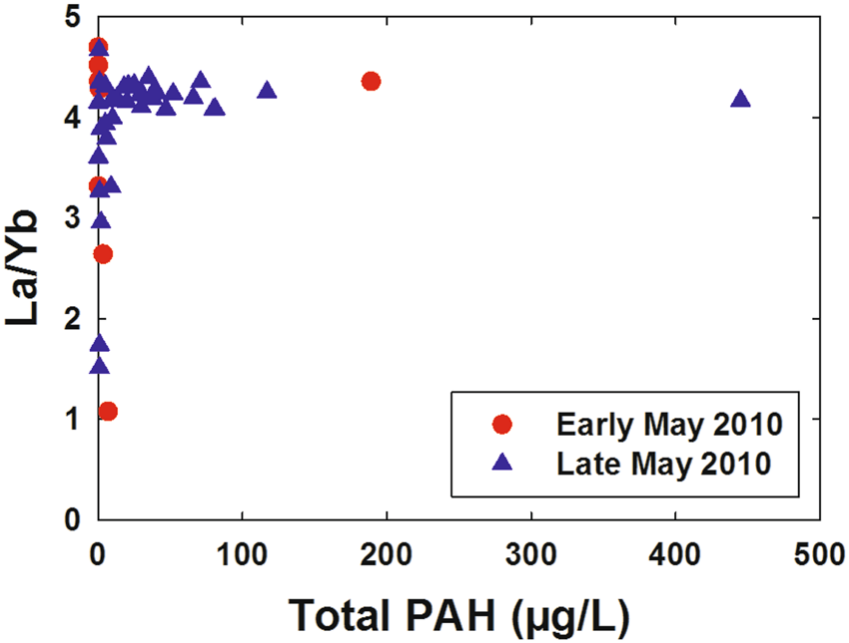
Dissolved La/Yb molar ratio versus total polycyclic aromatic hydrocarbons (PAHs) in deep water samples near the *Deepwater Horizon* site.

Association of the LREE depletion with crude oil rather than methanotrophy (either through biological respiration or some other means such as complexation and immobilization) seems unlikely. LREE-depleted samples had low concentrations of polycyclic aromatic hydrocarbons (PAHs), which were the oil component measured in these samples. Conversely, samples with high PAH concentrations had normal La/Yb ratios (Fig. 4).

**Figure 5:**
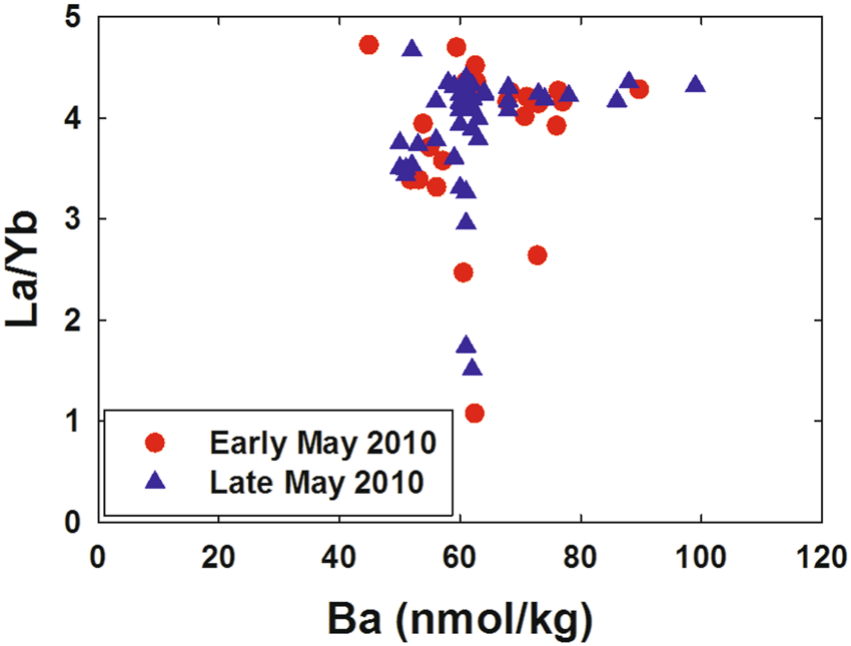
Dissolved La/Yb molar ratio versus dissolved barium in deep water samples near the *Deepwater Horizon* site.

**Figure 6:**
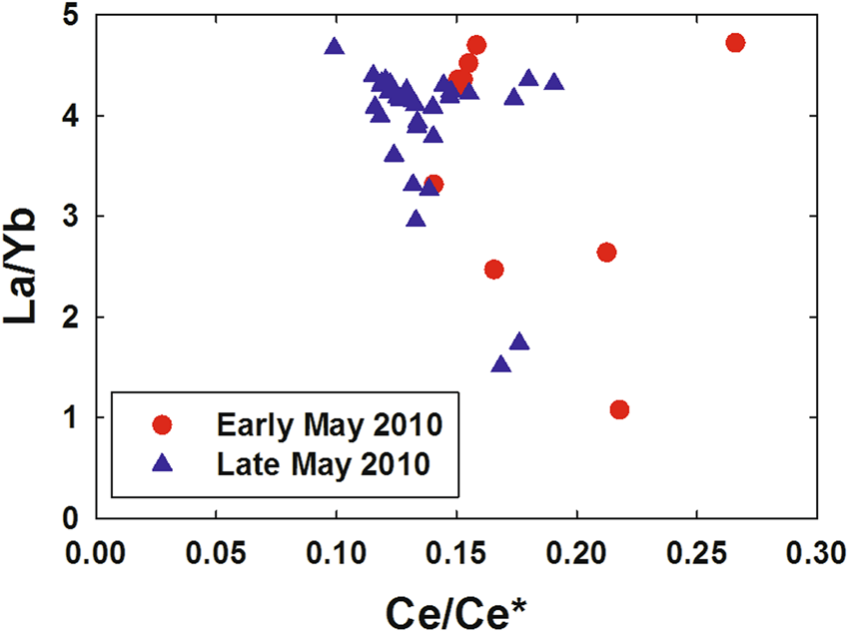
Dissolved La/Yb molar ratio versus the cerium anomaly (Ce/Ce*) in deep water samples near the *Deepwater Horizon* site. The Ce anomaly was calculated using La, Ce, and Pr concentrations normalized to North American Shale Composite^64^.

We also saw no relationship between LREE depletion and increased dissolved Ba (Fig. 5), which was associated with drilling muds used in the “top kill” attempt to control the blowout^15^; the “top kill” being a method in which drilling muds and other heavy materials are pumped into a leaking well to stop the flow of oil and gas. Specifically, samples with high dissolved Ba relative to background levels (~60 nmol/kg) tended to have background La/Yb ratios (~4.4) while samples with low La/Yb ratios tended to have background dissolved Ba concentrations. Thus, adsorption of the LREEs onto top kill materials and removal by settling seems unlikely. We also note that the Ce-anomaly was generally slightly higher in LREE-depleted samples as compared with most plume depth samples that were not LREE-depleted (Fig. 6). The Ce-anomaly (the ratio of measured Ce to the Ce concentration predicted from the concentrations of La and Pr) results from the tendency of oxidized Ce(IV) to be more readily scavenged by particles than its +III oxidation state REE neighbors^26, 27^. Because increased adsorptive removal of the LREEs would be expected to decrease the Ce-anomaly, the observed increase also argues against the LREE depletions resulting from particle adsorption. Most likely, the increased Ce-anomaly in LREE-depleted samples resulted simply from the greater methanotrophic removal of La relative to Ce (Fig. 2). We also note that limited experimental evidence suggests that REE adsorption onto bacterial cell walls is either flat across the series or results in preferential adsorption of heavy REEs^31–33^. Thus, adsorption onto cells of the microbial bloom is unlikely to have resulted in the REE patterns we observed during the DWH blowout.

We thus conclude that LREE depletion in the submerged *DWH* oil/gas plumes most likely resulted from uptake by methanotrophic organisms. Given recent reports of the ubiquity in marine environments of the LREE-containing XoxF MDHs and the genes that encode them^22–24^ along with the low concentrations of REEs in seawater, the LREE depletions we observed in *DWH* submerged methane plume waters should not be surprising.

**Figure 7:**
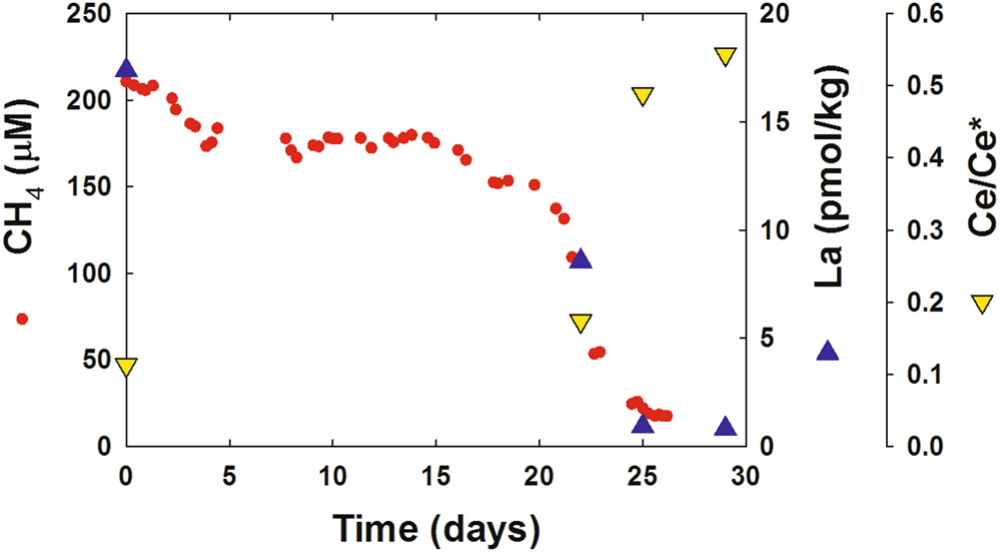
Results from an incubation experiment assessing the chemical kinetics of aerobic methane oxidation. Seawater was sampled in the northern Gulf of Mexico at the “Sleeping Dragon” seep (MC118; 28°51.129′N, 88° 29.51′W) in April 2015. Red circles: Dissolved methane concentration. Blue triangles: Dissolved lanthanum concentration. Yellow inverted triangles: cerium anomaly.

To supplement the field observations, four incubation experiments utilizing gas-tight, trace element clean mesocosms^34^ were conducted with waters collected at the MC118 methane seep site in the northern Gulf of Mexico, proximal to the DWH release site, in April 2015. Since the samples were collected immediately adjacent to a natural seep, the water had naturally high levels of dissolved methane. The results obtained from these incubation experiments displayed two notable characteristics. First, a substantial lag phase was observed. On average, the methane oxidation rates were relatively low for the initial 14 days (range 9–19 days) of incubation (Fig. 7). This effect likely resulted from the natural inoculation of ambient seawater with seep methane immediately before sampling in the natural environment and thus required time for the cell density to increase in response to the elevated methane load. However, after approximately day 14, the rates of oxidation increased substantially, reaching a first-order reaction rate constant of 0.22 day^−1^ on average (range 0.11 to 0.36 day^−1^). Following this increase in oxidation rates, methane was oxidized to near completion within 7.5 days on average (range 6 to 11 days) (Fig. 7). Second, we also observed a decrease in LREEs coincident with the decrease in methane. The average molar ratio of methane to La removal was 5.3 ± 3.3 × 10^6^ to 1. Since the observed decrease in La during the *DWH* incident was 4 to 12 pM (Fig. 1), this suggests that 8 to 103 µM of methane were removed. This amount of methane oxidation is in agreement with the amount of methane dissolved in these deep plumes^5, 7, 10, 13^, and suggests that *DWH* methane may have been oxidized completely and not limited by LREEs.

Two other key observations of the mesocosm incubations are that a) at the point in the experiments where we first noted significant REE drawdown, removal of La was greater than removal of other REEs and b) throughout the experiments the Ce-anomaly increased in all the mesocosms (Fig. 7). While these two observations are consistent with the preferential use of La in XoxF MDHs^20, 21^, they are at odds with experimental evidence relating to REE sorption on cell surfaces^31–33^ and marine suspended material^26, 27^. Note that killed controls were not performed as part of our mesocosm experiments. However, killed controls would not have adequately tested for the possibility of adsorptive removal of LREEs, as previous work^35^ indicates that commonly used microbial controls can interfere with abiotic as well as biotic metal-particle interactions. Additionally, since these were mesocosm incubations of natural samples rather than pure culture incubations, performing killed controls would have required our knowing what population would bloom and adding the appropriate distribution of killed but intact cells to the control mesocosms: a challenging proposition. Nonetheless, the synchronous draw down of methane and LREEs during the mesocosm incubations in a pattern consistent XoxF MDH utilization suggests that LREEs were biologically utilized rather than adsorbed to the surface of particles or cells.

Metagenomic sequencing of samples from the incubation experiments also showed that genes encoding LREE-containing XoxF MDHs were present, with sequences most closely related to those from marine *Methylococcaceae* (Figure S1 and Table S1). Given that the MC118 seeps are only 17 km northwest of the *DWH* blowout site and in water of 800–900 m depth, it seems likely that XoxF MDHs were also present in the closely related *Methylococcaceae* identified by Kessler *et al*.^10^ in the *DWH* submerged gas plumes.

Because of the limited spatial/temporal coverage of our field measurements, our work cannot completely answer the important question of whether LREE availability limited methanotrophy during the *DWH* blowout, though we suspect it did not. In lab experiments, Vu *et al*.^36^ did find that La^3+^ concentrations as low as 2.5 nM allowed growth of *Methylobacterium extorquens* AM1, albeit at a reduced rate. However, their experiments contained far higher (by >100-fold) LREE concentrations than observed in seawater and are thus difficult to put in the context of our observations. As discussed above, the results of our incubation experiments suggest that seawater at the *DWH* site contained an adequate supply of LREEs. Also, reported oxygen depletions throughout the blowout period rarely exceeded 25% of ambient concentrations^10, 11, 37^. This is similar to the highest amount of oxygen depletion we observed in our *DWH* samples; and, in our highest oxygen-depleted samples, the sum of the four LREEs was only about 40% depleted. Additionally, depletion of LREEs does not preclude methanotrophy by organisms utilizing Ca^2+^-dependent MDH. *mxaF* genes were also detected in our metagenome samples and were most closely related to those from the same group of marine *Methylococcaceae* with closely related *xoxF* genes (Figure S1); it is likely that the methanotrophs in our samples had both *xoxF* and *mxaF* genes. However, increased expression of the Ca^2+^-dependent MDH in methanotrophs due to LREE depletion has been shown to increase excretion of methanol, increasing the growth of non-methanotrophic methylotrophs that oxidize methanol^28^. Non-methanotrophic methylotrophs from the genus *Methylophaga* and family *Methylophilaceae* were abundant during the *DWH* spill^10^, but their role in methane oxidation has been debated^38^. Methanol excretion due to LREE depletion may explain why these methylotrophs were so abundant, despite their inability to directly oxidize methane, and may have slowed the growth of methanotrophs.

It is interesting that some marine methanotrophs use LREEs despite their seawater concentrations being more than 10^8^ times lower than dissolved Ca. Methanotrophy may be one of the oldest biochemical pathways^39^ and the LREE requirement for at least some methanotrophs suggests its origin is likely to have been in an environment comparatively rich in these elements such as the hydrothermal system studied by Pol *et al*.^20^ where LREE concentrations are ~4 × 10^4^ times greater than in the seawater near the *DWH* site. Given that hydrothermal waters often contain various light alkanes including methane, ethane, and propane (e.g., ref. 40), examination of other alcohol dehydrogenases for LREE requirements might prove a fruitful avenue of investigation. In fact, an ethanol dehydrogenase in *Methylobacterium extorquens* AM1 was recently determined to require La^41^. Related *exaA* alcohol dehydrogenase sequences were also identified in our incubation metagenomes (Figure S1 and Table S1). Many are closely related to sequences from *Colwellia* and *Cycloclasticus* species implicated in ethane and propane oxidation and shown to be highly expressed during the *DWH* spill^12, 42^. These may have also contributed to LREE depletion. However, methane was much more abundant than ethane and propane^7^, and methanotrophy was likely responsible for the majority of LREE depletion.

If LREE-utilizing methanotrophs are as widespread in the ocean as implied by the ubiquity in marine environments of XoxF MDHs and the genes that encode them^22–24^, then the distributions of dissolved REEs might well be affected in other methane-rich systems. We consider first the large methane releases from oceanic hydrate reservoirs that are thought to have been a possible contributor to past and perhaps future climate change^29, 43^. Using a 35 pM average oceanic concentration of La^44^ (the most abundant of the LREEs), yields an oceanic La reservoir of ~4.8 × 10^10^ mol. Our incubation experiments suggest this provides the capacity to oxidize ~1.0 to 4.1 × 10^17^ mol CH_4_ via the XoxF MDH pathway. This is similar in magnitude, though at the high end, of estimated methane releases during past hyperthermal periods (e.g., ref. 29). While intriguing, we note that our calculation ignores several important factors including possible oceanic recycling of LREEs and the rate of methanotrophy relative to the LREE residence time, which is estimated to be 500 yrs^45, 46^. Factoring in the La residence time suggests an equivalent rate of methane oxidation via XoxF MDH of 1.9 to 8.3 × 10^14^ mol CH_4_/yr. The lower bound of this estimate is equivalent to the upper estimates of seafloor methane release both today and 56 Ma ago during the Paleocene–Eocene Thermal Maximum (PETM)^43^. Taken together, these crude calculations suggest that for oceanic methanotrophy to significantly affect LREE distributions or inventories (or vice versa) would require either a massive methane release during a very short time span (i.e., <500 yrs) or localized regions of high methane input such as the *DWH* submerged methane plume. Determination of past oceanic inventory changes might be possible via determination of LREEs in buried foraminiferal carbonate.

Other localized regions of high methane input include pore waters in continental margin sediments as well as waters near cold seeps. Indeed, Bayon *et al*.^47^ observed slight depletion of dissolved Nd and enrichment in particulate Nd in methane-enriched plumes above cold seeps on the Niger Delta margin. They suggested this could be the result of REE scavenging on Fe/Mn oxides, but our results suggest biological uptake as an alternative explanation. Whether methanotrophy is of importance to REE distributions in low-methane open ocean waters is less clear. On the one hand, reported rates of methane oxidation in the open ocean are quite low (e.g., ref. 48) relative to both the aftermath of the *DWH* blowout and our incubation experiments. However, a consistent LREE depletion at the depth of the deep chlorophyll maximum (DCM) has been observed in sections across both the North and South Atlantic^49, 50^. Given that the DCM is often the locus of a methane maximum (e.g., ref. 51), investigation of the possible role of methanotrophy in overall oceanic REE cycling seems warranted. Furthermore, other biological stimulatory effects of REEs have previously been noted^52, 53^; thus, methanotrophy could well be only part of the story of the biogeochemistry of lanthanides.

## Methods

### Water sampling in the vicinity of *DWH*

Water sampling in the vicinity of the *Deepwater Horizon* blowout was conducted aboard the R/V *Pelican* (10–14 May 2010), R/V *F*.*G*. *Walton Smith* (26 May–1 June 2010), and R/V *Cape Hatteras* (20–29 Oct. 2011). During the first two cruises, samples were collected during multiple hydrocasts in a southwesterly trend within 27 km of the wellhead. During October 2011, samples were also collected during multiple hydrocasts, this time in all directions and ranging to over 100 km away from the wellhead.

Water samples were collected from rosette-mounted, Teflon-coated Go-Flo or external spring Niskin bottles (General Oceanics) that were precleaned using dilute acid (10% HCl), ultrapure water, an EDTA (10 mM) solution, and a final rinse with ultrapure water. Sampling depths were determined by examination of the *in situ* sensor profiles, paying particular attention to anomalies in dissolved oxygen (DO), light transmission, and CDOM fluorescence. Because of the short notice with which the first cruise was arranged, clean Go-Flo or Niskin bottles were not available. For that cruise, the ship’s normal rosette-mounted Niskin bottles were used. Samples were cleanly filtered using 0.45 µm polyethylene syringe filters^54^.

Station locations, hydrocast data (nutrients, oxygen, PAHs, and dissolved metals except the LREE data described here), and details of sampling and shipboard sample processing can be found in previous reports dealing with macro-nutrient^14^ and trace element distributions^15^ in the vicinity of the *DWH* wellhead.

### Incubation experiments

In order to more carefully observe the relationship between aerobic methane oxidation in the waters of the northern Gulf of Mexico and the removal of LREEs, four mesocosm incubation experiments were conducted. Seawater was sampled from the 12–17 April 2015 on the E/V *Nautilus* using the ROV *Hercules*. An *in-situ* seawater pumping device (SUPR sampler; ref. 55) was attached to the arm of the ROV *Hercules* enabling seawater to be precisely pumped into custom seawater incubation bags^34^. This protocol enabled the collection of seawater immediately adjacent to natural methane bubble emissions from the “Sleeping Dragon” seep (MC118; 28°51.129′N, 88° 29.51′W). As such, these seawater incubation samples began with dissolved methane concentrations ranging from 47 to 172 μM. The incubation bags, which were secured to the ROV *Hercules*, were removed upon recovery of the ROV and transferred into a 6 °C incubator. In addition, the seawater incubation bags were attached to a custom designed seawater analysis system^34^. This system is designed to collect, store, and incubate seawater samples, periodically analyzes the water for the dissolved concentrations of methane, carbon dioxide, and oxygen, and has proven to not contaminate seawater samples with nutrients or trace metals as well as be gas impermeable over the incubation time scale (i.e., months; ref. 34). These specific samples were incubated for up to 29 days and changes in methane, carbon dioxide, and dissolved oxygen were recorded. Samples were periodically removed from the incubation bags for the analysis of trace metals, LREEs, and DNA.

Blank water and dissolved methane standards were stored in separate incubation bags and analyzed over time for dissolved methane and oxygen concentrations as well as LREE concentrations. No change in dissolved gases was observed during these tests and the trace metal concentrations also were not significantly changed by these incubation protocols^34^.

### Metagenomic Sequencing and Analysis

Over the course of the incubations, DNA was collected by filtration of 1 L seawater through a 0.22 µm Sterivex filter (Millipore). DNA was extracted from filters with the Fast DNA SPIN Kit for Soil (MP Biomedicals) and two samples from one of the bags were selected for metagenomic sequencing. Sequencing was performed at the DOE Joint Genome Institute (JGI) using the Illumina HiSeq. 2500 system and standard JGI protocols for assembly with MEGAHIT v1.0.3^56^ and annotation with the DOE-JGI Metagenome Annotation Pipeline (version 4.10.2)^57^. Metagenome sequences and data are available through IMG under IMG genome IDs 3300008227and 3300008252. Methanol and related alcohol dehydrogenase sequences in assembled metagenomes were identified by BLAST searches with reference *mxaF* and *xoxF* sequences from the phylogenies shown in Keltjens *et al*.^21^ (Table S1). Phylogenetic analysis of all metagenome sequences of sufficient length (>180 amino acids), their closest sequence matches in IMG, and a subset of the sequences analyzed by Taubert *et al*.^23^ was conducted with MEGA 7.0^58^. *mxaF* MDH sequences (KO:K14028) were annotated accurately by the IMG annotation pipeline, as were *exa*A alcohol dehydrogenases (KO:K00114). *xoxF* MDHs were all annotated as glucose dehydrogenase, but predominately clustered near *xoxF* sequences from marine *Methylococcaceae* (Figure S1). *mxaF* sequences were also most closely related to those from the marine *Methylococcaceae* (Figure S1). Both *xoxF* and *mxaF* sequences showed relatively low diversity, with nearly all sequences clustering together, so only 3 of each are shown in Figure S1.

### Trace element analysis

For analysis of dissolved rare earth elements (including Y), 7 mL of sample was spiked with a mixture of isotopically-enriched Nd-145, Sm-149, Eu-153, Gd-155, Dy-161, Er-167, and Yb-171 (Oak Ridge Nat’l. Labs). Each spike was >90% enriched in the listed isotopes. The sample/spike ratio was chosen so as to have the analytical isotope ratios approximately the geometric mean of the natural and enriched spike isotope ratios. Samples were then extracted/pre-concentrated using a SeaFAST system (Elemental Scientific, Inc.) operated in offline mode. A similar online SeaFAST extraction procedure is described by Hathorne *et al*.^59^. The extracted samples were subsequently analyzed using a Thermo-Fisher high resolution ICP-MS with an Apex-FAST high efficiency sample introduction system including a Spiro desolvator (Elemental Scientific, Inc.). The instrument was operated in low resolution. The enriched isotope spikes also served to provide counts/sec. calibration factors for elements that were not spiked with enriched isotopes. This calibration was also examined with a standard made in dilute nitric acid. Precision and recovery were checked by analysis of a large-volume composite North Atlantic surface seawater sample. Spiked (with a natural isotopic abundance elemental spike) and unspiked aliquots of this sample were analyzed twice in each analytical run. A Ba standard was also run to check for BaO^+^ interference on several isotopes and Ba in the extracted samples was also monitored. Because the extraction resin in the SeaFAST system (Nobias PA-1) discriminates against Ba, plus the reduction of the BaO^+^ interference by the desolvation system, BaO^+^ was less than 0.1% of the counts in Eu-151, Eu-153, Gd-155, and Gd-157. Tests also revealed no significant low REE oxide interference on mid-/high-REEs.

Application of this analytical method to 14-mL sample aliquots from a profile collected at the Bermuda Atlantic Times Series station (BATS), yielded results equivalent to those previously published for that station by van de Flierdt *et al*.^60^, Pahnke *et al*.^61^, and Middag *et al*.^62^. Detection limits were typically <1% of the concentrations reported here except for Ce and Eu where detection limits were <5% of the reported concentrations. Precision (1σ) was typically ±2% and recoveries were typically 102 ± 3%.

Determination of dissolved Ba followed the isotope dilution, high resolution ICP-MS methodology described in Shim *et al*.^63^.

### Data availability

Data are available from the corresponding author or through the Gulf of Mexico Research Initiative Information & Data Cooperative (GRIIDC) at https://data.gulfresearchinitiative.org; doi:10.7266/N7MS3QQ5; doi:10.7266/N7CC0XQ6; doi:10.7266/N7RR1WPX. Metagenomic data are available through the DOE Joint Genome Institute’s Integrated Microbial Genomes & Microbiomes (IMG/M) platform at https://img.jgi.doe.gov/m, datasets 3300008252 and 3300008227.

## Electronic supplementary material

Supplementary Information
