# Supplementary material for: Light rare earth element depletion during Deepwater Horizon blowout methanotrophy
Source: Sci Rep. 2017 Sep 4;7:10389. doi: 10.1038/s41598-017-11060-z (PMC5583346; doi:10.1038/s41598-017-11060-z)
Supplement: Supplementary file 1 — Supplementary Information [file 41598_2017_11060_MOESM1_ESM.pdf]

Light rare earth element depletion during *Deepwater Horizon* blowout methanotrophy

Authors: A.M. Shiller[1*], E.W. Chan[2], DJ. Joung[1], M.C. Redmond[3], J.D. Kessler[2]

[1]Center for Trace Analysis, University of Southern Mississippi, Stennis Space Center, MS 39529, USA.

[2]Earth and Environmental Sciences, University of Rochester, Rochester, NY 14627, USA.

[3]Dept. of Biological Sciences, University of North Carolina at Charlotte, Charlotte, NC 28223, USA.

*Correspondence to: alan.shiller@usm.edu; 228-688-1178

Supplementary Information: Table S1 and Figure S1.

Table S1. Summary of metagenome sequences

Sample Name	**GOM15B**	**GOM15C**
IMG ID	3300008252	3300008227
Genome Size	802,871,420	845,437,039
Gene Count	1,680,640	1,822,033
Scaffold Count	1,244,072	1,379,518
%GC	42.5	41.8
16S rRNA Gene Count	1252	1304
16S rRNA Reads	268,350	290,117
xoxF Gene Count	125	184
xoxF Reads	10,307	11,247
mxaF Gene Count	46	46
mxaF Reads	12,225	11,130
exaA Gene Count	220	206
exaA Reads	13,396	15,615

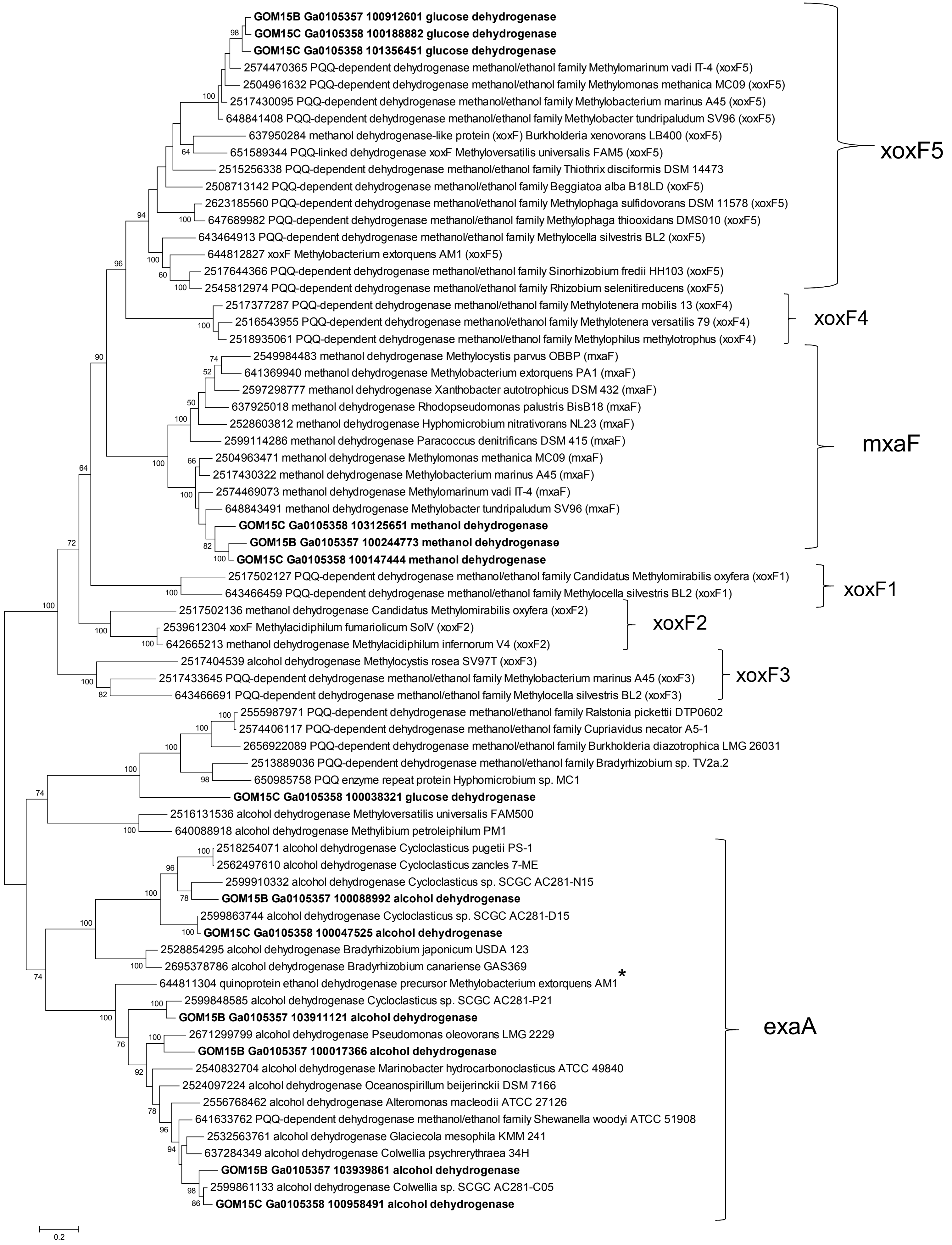

Figure S1: Maximum likelihood tree of mxaF and xoxF methanol dehydrogenase and exaA alcohol dehydrogenase sequences. Selected sequences from incubation metagenomes are indicated in bold and identified by IMG locus tags. La-requiring exaA from Methylobacterium extorquens AM1 is shown with *. Reference sequences are shown with IMG gene IDs and annotations. xoxF sequences are also classified according to the five clade system used by Keltjens et al. (2014), shown in parentheses. Bootstrap values below 50% are not shown.
